# C-terminal Domain of the Heavy Chain of Tetanus Toxin Ameliorate Lipopolysaccharide Induced Hemiparkinsonism in Rats

**DOI:** 10.1007/s12640-026-00811-4

**Published:** 2026-07-18

**Authors:** Irving Parra, José Aguilera, Yousef Tizabi, Liliana Mendieta

**Affiliations:** 1 Laboratorio de Neuroquímica, Facultad de Ciencias Químicas, Benemérita Universidad Autónoma de Puebla, 14 Sur y Av. San Claudio CU, Col. San Manuel, Puebla, 72592 México; 2 Departament de Bioquímica i Biologia Molecular, Institut de Neurociències, Universitat Autònoma de Barcelona, Cerdanyola del Vallès, Barcelona, 08193 España; 3 Centro de Investigación Biomédica en Red Enfermedades Neurodegenerativas (CIBERNED), Instituto de Salud Carlos III, Madrid, 28031 España; 4 Department of Pharmacology, Howard University College of Medicine, Washington, DC USA

**Keywords:** Neuroinflammation, Lipopolysaccharide, Tetanus toxin, Astroglia, Parkinson’s disease model, Rat

## Abstract

Neuroinflammation is recognized as a central mechanism in Parkinson’s disease (PD) pathogenesis. The C-terminal domain of the heavy-chain of tetanus toxin (Hc-TeTx) has shown neuroprotective effects in toxin-based PD models, but its efficacy in inflammatory-induced PD conditions remain unexplored. To test this possibility, we used the hemiparkinsonism model induced by central injection of lipopolysaccharide (LPS) to evaluate potential antiinflammatory effects of Hc-TeTx in adult male Wistar rats. Seven days post LPS-injection motor coordination and gait maintenance were assessed using elevated beam test. The day after, that is 8 days post LPS-injection, the cylinder test was used to assess forelimb motor asymmetry during spontaneous vertical exploration. Immediately after this test the animals were sacrificed for tissue collection. Dopaminergic degeneration was assessed by immunohistochemical quantification of tyrosine hydroxylase in *substantia nigra pars compacta*. Astroglial activation, reflective of neuroinflammation was evaluated by glial fibrillary acidic protein in the striatum. LPS administration caused forelimb motor asymmetry, which was significantly attenuated by Hc-TeTx treatment. Hc-TeTx also tended to attenuate LPS-induced dopaminergic neurodegeneration reflected by a reduced TH ipsilateral/contralateral ratio, as well as astroglial activation that was markedly increased after LPS administration. Beam test parameters showed no significant differences among groups. Although further verification and mechanistic studies are needed, current results support potential utility of Hc-TeTx in an inflammatory model of PD.

## Introduction

Parkinson’s disease (PD) is a progressive neurodegenerative disorder characterized primarily by the degeneration of dopaminergic neurons in the *substantia nigra pars compacta* (*SNpc*) and the consequent disruption of the nigrostriatal pathway. This neuronal loss leads to the cardinal motor symptoms of the disease (Poewe et al. 2017; Heinzel et al. 2019; Jankovic and Tan 2020). Although the exact etiology of PD remains elusive, increasing evidence indicates that neuroinflammation plays a critical role in both the initiation and progression of the disease (McGeer et al. 1988; Hirsch and Hunot 2009; Castillo-Rangel et al. 2023; Chen et al. 2026). Activated microglia and astrocytes release proinflammatory cytokines, reactive oxygen species, and other cytotoxic mediators that contribute to mitochondrial dysfunction, oxidative stress, and neuronal injury, thereby amplifying neurodegenerative processes (Pajares et al. 2020; Muzio et al. 2021; Chen et al. 2023; Jurcau et al. 2023; Domínguez Rojo et al. 2024; Wang 2025).

Experimental models using intracerebral administration of lipopolysaccharide (LPS), a potent inducer of innate immune activation, have provided valuable insights into the role of inflammation in PD pathogenesis (Castaño et al. 1998; Flores-Martinez et al. 2018; Valenzuela-Arzeta et al. 2023; Casanova et al. 2026). Intrastriatal LPS injection induces an ongoing inflammatory response that propagates retrogradely to *substantia nigra* (*SN*), resulting in gradual dopaminergic neuron loss and motor deficits (Choi et al. 2009; Hunter et al. 2017; Parra et al. 2020). Unlike acute neurotoxin models such as 6-hydroxydopamine (6-OHDA), 1-methyl-4-phenyl-1,2,3,6-tetrahydropyridine (MPTP) or 1-methyl-4-phenylpyridinium (MPP^+^), which produce rapid and extensive neuronal death through direct oxidative and mitochondrial toxicity, the LPS model produces a continuous neurodegenerative process driven by sustained inflammatory signaling (Parra et al. 2020). This progressive pattern more closely resembles early pathogenic mechanisms observed in PD, where neuronal dysfunction precedes overt cell death and clinical symptoms (Liu and Bing 2011; Casanova et al. 2026).

Among candidate neuroprotective molecules, the recombinant C-terminal domain of the heavy-chain of tetanus toxin (Hc-TeTx) has emerged as a promising neuroprotective agent. Hc-TeTx binds selectively to neuronal membranes and undergoes retrograde axonal transport to neuronal cell bodies, where it activates intracellular survival pathways that promote neuronal resistance to stress and injury (Gil et al. 2000, 2003; Calvo et al. 2012; Ovsepian et al. 2016). Previous studies have demonstrated that Hc-TeTx protects dopaminergic neurons and improves motor function in toxin-based models of PD (Mendieta et al. 2009; Patricio et al. 2019), Alzheimer’s disease (Patricio-Martínez et al. 2016), amyotrophic lateral sclerosis (Moreno-Martinez et al. 2020), and methamphetamine toxicity (Mendieta et al. 2016). Moreover, Hc-TeTx showed sustained antidepressant effects in an animal model of depression, a condition commonly co-morbid with PD (Hurley and Tizabi 2013; Getachew et al. 2019; Tizabi et al. 2021; Bracone et al. 2026). The neuroprotective effects of Hc-TeTx have been attributed to activation of Trk receptors induced-pro-survival signaling pathways and anti-apoptotic signaling that enhance neuronal resilience to oxidative and metabolic stress (Gil et al. 2003; Chaïb-Oukadour et al. 2004, 2009; Moreno-Galarza et al. 2018; Candalija et al. 2021).

However, despite growing evidence supporting the neuroprotective properties of Hc-TeTx, its efficacy in inflammation-driven neurodegeneration remains largely unexplored (Mendieta et al. 2012, 2016; Getachew et al. 2019; Patricio et al. 2019, 2022; Moreno-Martinez et al. 2020). Since neuroinflammation is strongly implicated in initiating and sustaining dopaminergic neuronal loss (Hirsch and Hunot 2009; Castillo-Rangel et al. 2023; Chen et al. 2026), therapeutic strategies targeting early inflammatory stages may offer greater translational potential (Hirsch and Hunot 2009). Therefore, the present study was conceived as an initial proof-of-concept to determine whether Hc-TeTx could confer neuroprotection in an inflammation-driven model of PD.

## Methodology

### Animal Housing

Adult male Wistar rats (*Rattus norvegicus*; body weight 291 ± 18 g at the time of surgery; *N* = 44) were used in this study. Rats were purchased from a breeding colony of the *Benemérita Universidad Autónoma de Puebla (BUAP)* and raised on our facilities. Animals were housed in polycarbonate cages under controlled environmental conditions (temperature 23 ± 2 °C; relative humidity 48 ± 8%) and maintained on a 12 h light/dark cycle (lights on at 07:00 h), with *ad libitum* access to food and water. Experimental procedures were reviewed and approved by the *Comité para el Cuidado y Uso de los Animales de Laboratorio* (CCUAL) of the *BUAP*, and all animal protocols were conducted in accordance with the *Norma Oficial Mexicana *(NOM-062-−1999). Every effort was made to minimize animal suffering (*Guide for the care and use of laboratory animals*, 2011). Methodological reporting and experimental design were conducted according to ARRIVE 2.0 recommendations (Percie du Sert et al. 2020).

Upon arrival at the vivarium, animals were randomly assigned an identification number and weighed. Rats of similar body weight were group-housed (initially, 4 animals per cage), which was randomly adjusted to minimum of 2 per cage as they gained weight to maintain appropriate housing conditions before to start experiments. To promote environmental enrichment, reduce stress, and enhance social habituation, animals were gently handled for few mins each day and weighed every other day. In addition, rats were periodically placed together in a large enrichment arena containing bedding material and toys, allowing free social interaction under supervised conditions. The animals were habituated to the behavior room for 30 min prior to the behavioral tests. These procedures facilitated socialization, exploratory behavior, and habituation to experimental handling, thereby minimizing stress prior to surgical intervention and behavioral assessments.

**Fig. 1:**
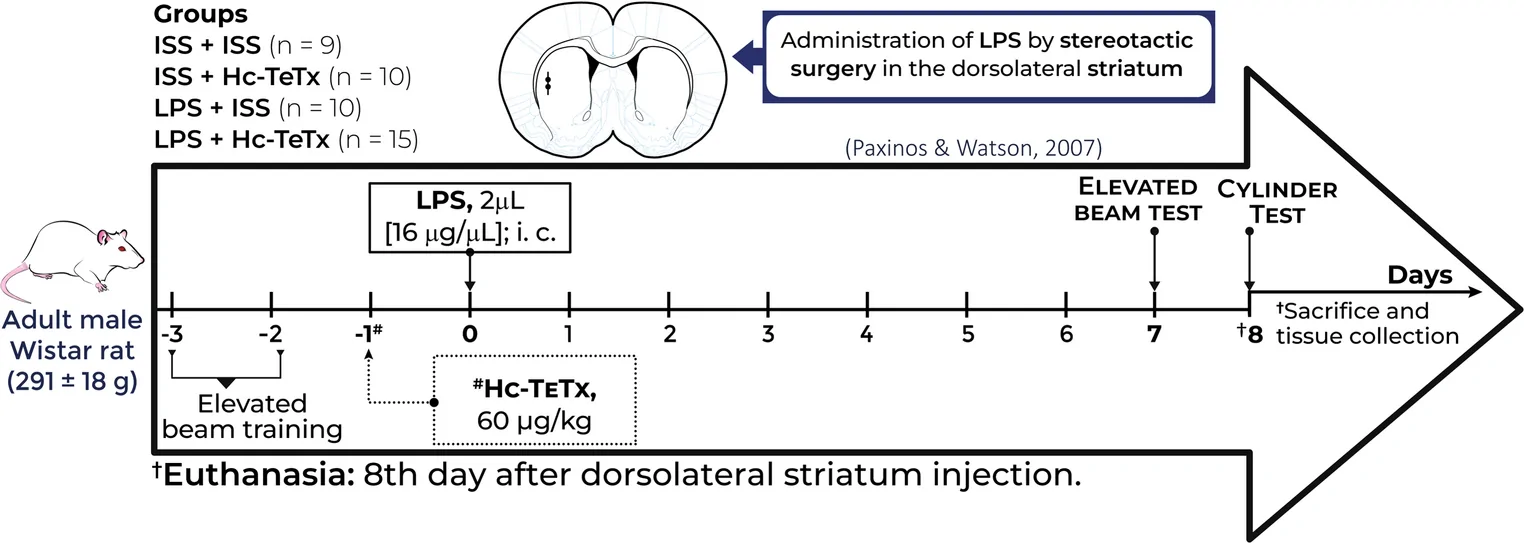
Timeline of the experimental design. Intracerebral injections of lipopolysaccharide (LPS) were performed in the left dorsolateral striatum of male rats (Rattus norvegicus; 291 ± 18 g at the time of surgery; *N* = 44) according to Paxinos and Watson (2014) coordinates. Experimental subjects performing behavioral test as Cylinder Test and Beam Walking Test to evaluated forelimb motor asymmetry during spontaneous vertical exploration and gait parameters and motor coordination, respectively. Animals were euthanized 8 days after surgery. Brains was processed for immunohistochemistry. Hemi-parkinsonian model induction: LPS [16 µg/µL] or LPS’ vehicle (isotonic saline solution, ISS) was injected stereotactically on two dorsoventral coordinates each one on striatum at AP: +0.8 mm; ML: +3.8 mm; DV1: −4.9 mm; DV2: −5.3 mm. Treatment regimen: Recombinant C terminal domain of heavy chain tetanus toxin (Hc-TeTx) or vehicle treatment ISS was administered one day before to stereotaxic surgery administered intraperitoneally. Abbreviations: Hc-TeTx, Recombinant C terminal domain of heavy chain of tetanus toxin; i.c, intracerebral administration; ISS, isotonic saline solution; LPS, lipopolysaccharide

Following stereotaxic surgery, animals were housed individually in standard polycarbonate cages (38 cm × 24 cm × 20 cm) to prevent injury and ensure accurate postoperative monitoring (Fig. 1).

### Experimental Design

#### Group Designation

Sample sizes were determined based on previous studies using similar experimental paradigms and expected variability in behavioral and histological outcomes (Parra et al. 2020, 2023). The experimental unit was defined as an individual animal because animals was housed individually in a home cage, and treatments were administered and outcomes measured independently for each subject.

Animal were randomly selected to assign to one of follow groups:

- Isotonic Saline Solution (ISS), intrastrial + ISS, intraperitoneal (i.p.) [ISS + ISS; *n* = 9].
- ISS, i.c. + Hc-TeTx, i.p. [*n* = 10]
- LPS, i.c. + ISS, i.p. [*n* = 10]
- LPS, i.c. + Hc-TeTx, i.p. [*n* = 15]

#### Stereotactic Surgery Procedures

Prior to surgery, animals were anesthetized with a mixture of ketamine [115.2 mg/ml] and xylazine [20 mg/ml] (90:9 mg/kg; i.p.) (Dyson et al. 2023; Oh and Narver 2024). LPS extracted from *Escherichia coli* serotype O26:B6 (≥ 10,000 EU/mg, purified by phenol extraction; Cat. No. L8274; Sigma-Aldrich, St. Louis, MO, USA), was administrated at concentration of 16 µg/µL, with 1 µL injected at each two dorsoventral (DV) coordinates in the left dorsolateral striatum with five minutes between injections (total injected volume: 2 µL; total dose: 32 µg per animal) according to our previous studies (Parra et al. 2020, 2023). The stereotaxic coordinates were AP: +0.7 mm respect to bregma; ML: +3.4 mm from midline; DV1: −4.8 mm and DV2: −5.2 mm below dura (Paxinos and Watson 2014). Additionally, as a control group, ISS [0.9%] was administered under the same conditions. The infusion rate for injection was 0.2 µL/min.

All animals received appropriate preoperative, intraoperative and postoperative care. Prior to skin incision, 100 µL of 1% lidocaine solution (10 mg/mL; PISA Laboratory, Mexico), was administered subcutaneously to induce local anesthesia. Systemic analgesics were intentionally avoided due to their potential interference with neuroinflammatory responses. During surgery, 0.5% hypromellose ophthalmic solution (AMSA Laboratories, Mexico) was applied to the cornea to maintain corneal lubrication. Additionally, the animals were placed on prewarmed pads throughout the surgical procedure to preserve body temperature. During recovery, animals were monitored for oxygen saturation and body temperature. Hydration was maintained by subcutaneous administration of 1 mL of Hartmann’s solution every hour beginning at anesthetic induction and continuing until complete outpatient recovery. Additionally, postoperative care included topical lidocaine (1%) spray (10 mg/mL; PISA Laboratory, Mexico) on the sutured wound site, gentle handling, and environmental enrichment. No animals died due to anesthetic or surgical procedures.

#### Treatment Procedure

Hc-TeTx was synthesized and lyophilized according to Herrando-Grabulosa and collaborators (Herrando-Grabulosa et al. 2020) at *Institut de Neurociències*,* Universitat Autònoma de Barcelona*. Hc-TeTx solution was prepared by dissolving 1 mg of lyophilized Hc-TeTx in 4 mL of saline solution. Animals received recombinant Hc-TeTx i.p. at a dose of 60 µg/kg, administered 24 h before surgery to allow adequate retrograde transport to the brain (Getachew et al. 2019; Mendieta et al. 2016; Moreno-Martinez et al. 2020).

#### Cylinder Test

Forelimb (FL) use asymmetry was evaluated 8 days after LPS injection, prior to euthanasia by cylinder test. Each animal was placed individually in a transparent acrylic cylinder (20 cm diameter × 30 cm height) and allowed to explore freely for 5 min. Behavior was recorded using a digital video camera positioned above the cylinder (30 frames per second (fps), 720p resolution). Video recordings were analyzed offline using Adobe Premiere Pro (version 2026; Adobe Inc., San Jose, CA, USA) for frame-by-frame temporal measurements to quantify the number of wall contacts performed with the left forelimb (LF, ipsilateral FL), right forelimb (RF, contralateral FL), or both forelimbs (BF) simultaneously during rearing.

Behavioral assessment was based in previously protocols (Liu et al. 1999; Schallert and Tillerson 2000; Schallert et al. 2000; Tillerson et al. 2001). Briefly, forelimb use was defined as a functional and discrete wall-contact event in which one or both forelimbs were placed against the cylinder wall while producing a change or maintenance in body support, center-of-mass control, vertical posture, or lateral weight transfer during a vertical exploratory bout. A forelimb use was considered functionally and scored as valid when at least one was observed: (i) full palmar contact against the cylinder wall; (ii) visible compression of the fingertip pads; or (iii) active extension of the digits onto the wall surface. Contacts made with the dorsal surface of the paw, wrist, or forearm, were excluded. Independent unilateral forelimb use (LF or RF) was scored when only one FL contacted the wall, or when the second forelimb contacted the wall after a forelag interval of > 0.4 s. Simultaneous forelimb use (BF) was recorded when both forelimbs contacted the cylinder wall in the same video frame or when the second forelimb contacted the wall within a forelag interval of ≤ 0.4 s after the first forelimb.

Data were collected manually on a datasheet and this data was used to calculate the laterality index (LI) according to the following formula:

$$\:LI=\frac{RF-LF}{RF+LF+BF}$$

where RF corresponds to right forelimb contacts, LF to left forelimb contacts, and BF to simultaneous use of both forelimbs (Oldfield 1971; Groneberg et al. 2025).

#### Elevated Balance Beam Test

Motor coordination and gait parameters were assessed using the elevated beam test 7 days after LPS injection. The apparatus consisted of a stainless-steel beam (100 cm long × 2 cm wide), connected to a start platform (20 cm × 4 cm) and a dark escape box (25 cm per side), elevated 100 cm above the floor (Allbutt and Henderson 2007; Parra et al. 2023). Animals underwent a training phase consisting of two consecutive days prior to surgery (days − 3 and − 2). During testing, rats were placed on the start platform and allowed to cross the beam voluntarily toward the escape box. Five effective crossings per animal were analyzed. Mean values were calculated from five successful crossings per animal. Each session was video-recorded using a digital camera (60 fps, 1080p resolution) for subsequent offline analysis. Video analysis was performed using Adobe Premiere Pro (version 2026; Adobe Inc., San Jose, CA, USA) for frame-by-frame temporal measurements and Fiji (an ImageJ distribution; National Institutes of Health, Bethesda, MD, USA) for spatial measurements. Data were collected on a digital datasheet.

The following parameters were quantified for both contralateral FL and hindlimbs (HL): Crossing latency (s), Mean speed (cm/s), Number of strides, Cadence (stride/s), Step cycle duration (s), Support time (%), Swing time (%), Stride length (cm), Stride error percentage, and Motor impairment.

*Latency* was defined as the time required to traverse the beam. *Mean speed* was calculated as beam length divided by latency. *Cadence* was defined as the number of strides per second. *Step cycle* duration was defined as the time required to complete one step cycle, consisting of support and swing phases. *Support time* was defined as the percentage of the step cycle during which the limb remained in contact with the beam, whereas *swing time* corresponded to the percentage of the step cycle during which the limb was not in contact with the beam. *Stride length* was defined as the distance between two consecutive paw placements of the same limb (Hamers et al. 2006; Koopmans et al. 2007; Baker 2013; Boix et al. 2018). *Stride error percentage* was defined the percentage of incorrect paw placements relative to the total number of strides. In addition, *Motor impairment* was defined as the relative proportion of each error subtype and was calculated as a fraction of the total number of incorrect paw placements (Brailowsky et al. 1986; Bueno-Nava et al. 2010; Avila-Luna et al. 2018; Parra et al. 2023).

*Motor impairment* was evaluated through *error type classification* using a modified version of the Brailowsky scale. Error type 2 from the original Brailowsky scale, described as hypotonia, was intentionally excluded from the present analysis. This decision was based on the inherently dynamic nature of hypotonia and its susceptibility to subjective interpretation during behavioral observation. Accurate assessment of hypotonia requires electrophysiological or biomechanical measurements, which were beyond the scope of the present study. Therefore, the scoring system was adjusted to include only objectively identifiable motor errors (See Fig. 3). Motor impairment was defined as, *none-error*: correct paw placement on the beam; *error type 1*: paw placed on the beam with partial toe displacement; *error type 2*: paw slips completely off the beam; *error type 3*: limb dragging or complete loss of postural support. Error classification and quantification were performed using frame-by-frame video analysis to ensure objective and reproducible assessment.

Gait parameters which are intrinsically dependent on locomotor speed, cadence, support time, swing time, step cycle duration, stride length, and swing speed (Clarke and Parker 1986; Batka et al. 2014; Aceves et al. 2020), were normalized by using a correction factor as described by Boix et al. (2018).

#### Immunohistochemistry (IHC)

Animals were deeply anesthetized with sodium pentobarbital (60 mg/kg, i.p.) (AVMA, 2020; Leary 2020) and intracardially perfused with paraformaldehyde (4%) in phosphate-buffered saline (PBS; 15 mM, pH 7.4). Brains were carefully removed and post-fixed in the same fixative solution for 15 days at 4 °C. Coronal brain Sect. (60 μm thickness) were obtained using a sliding microtome (Model SM2010R; Leica Microsystems, Wetzlar, Germany) and stored in PBS (15 mM, pH 7.4) containing sodium azide (0.1%) at 4 °C until processing.

Immunohistochemical staining was performed on free-floating sections using a standard avidin–biotin–peroxidase method. Sections were incubated for 10 min in PBS containing 2% Triton™ X-100 (Cat. No. X100-1 L; Sigma-Aldrich, St. Louis, MO, USA), 3% hydrogen peroxide to quench endogenous peroxidase activity, and 1% normal goat serum (Cat. No. 5425; Cell Signaling Technology, Danvers, MA, USA) to reduce nonspecific binding. They were then incubated for 18–24 h at 4 °C with the following primary antibodies: anti-Tyrosine Hydroxylase (TH) antibody (1:2000; rabbit polyclonal; Cat. No. AB152; Sigma-Aldrich, St. Louis, MO, USA), used as a marker of dopaminergic neurons, and anti-Glial Fibrillary Acidic Protein (GFAP) (D1F4Q) antibody (1:500; rabbit monoclonal; Cat. No. 12389; Cell Signaling Technology, Danvers, MA, USA), used as a marker of astroglial cells or astrocytosis. After washing in PBS, sections were incubated at room temperature with biotinylated goat anti-rabbit IgG (H + L) secondary antibody (1:500; Cat. No. BA-1000; Vector Laboratories, Burlingame, CA, USA), followed by streptavidin–horseradish peroxidase (HRP) conjugate (1:5000; Cat. No. 43–4323; Thermo Fisher Scientific, Waltham, MA, USA).

Immunoreactivity was visualized using 3,3′-diaminobenzidine tetrahydrochloride hydrate (DAB; 0.5 mg/mL; Cat. No. D5637-10 g; Sigma-Aldrich, St. Louis, MO, USA) as chromogen for 10 min. The reaction was stopped by rinsing in PBS. Sections were mounted onto 3% gelatin-coated slides, air-dried, dehydrated through ascending ethanol concentrations, cleared in xylene, and coverslipped using a permanent mounting medium. Negative control sections processed without primary antibodies were included to confirm staining specificity. Images were acquired using a bright-field microscope (Zeiss Axiolab 5; Zeiss, Oberkochen, Germany) under standard illumination conditions.

TH-positive (TH+) neurons in the *SNpc* were manually counted using the Cell Counter plugin in Fiji (an ImageJ distribution; National Institutes of Health, Bethesda, MD, USA). Micrographs of the *SNpc* (approximate − 5.0 from Bregma) were acquired using a 10× objective. Astrogliosis was quantified in the dorsolateral striatum (approximate − 0.8 from Bregma) by measuring the percentage of GFAP-positive (GFAP+) area using threshold-based segmentation in Fiji. Images for GFAP analysis were acquired using a 20× objective under identical illumination and acquisition settings. The GFAP+ area fraction was calculated as the percentage of immunoreactive area relative to the total analyzed field. To control for inter-individual variability and anatomical asymmetry, both TH+ neuron counts and GFAP+ area measurements were expressed as the ratio of the ipsilateral hemisphere (lesioned side) to the contralateral hemisphere (internal control). This normalization enabled quantification of dopaminergic neuronal loss and astroglial activation induced by LPS administration (Cuevas-Carbonell et al. 2022).

#### Randomization and Blissing Methods

Rat’s body weight was monitored every two days to assess health status and determine the optimal time point for surgery. Animals with similar body weights were scheduled for stereotaxic surgery to minimize variability associated with physiological differences. Surgeries were performed in a staggered manner, with four animals operated per session using a simple randomization procedure. Within each surgical session, a constrained randomization strategy was used to ensure balanced group representation. This approach ensured that each surgical session included representation from all experimental groups while maintaining random allocation. Researcher that performing stereotaxic surgeries and administering treatments was not blinded to group allocation during surgery. Therefore, surgery and behavioral testing was conducted in cohorts with four animals per session, so the order of behavioral testing was randomized by shuffling animal identification cards prior to each session to minimize order effects. Although complete blinding during surgical procedures was not feasible due to the investigator performing all experimental interventions, randomization procedures and delayed blinded outcome assessment were implemented to minimize potential sources of bias.

The same sense, as all animals were identified using numerical codes unrelated to treatment allocation, video recordings and histological samples were labeled exclusively with these identification numbers, and data analysis was conducted using coded identifiers without reference to treatment group. Behavioral videos and histological images were analyzed using a simple randomization procedure to ensure traceability while maintaining random allocation and minimizing potential bias during evaluation. To minimize bias during outcome assessment, behavioral, histological and statistical analyses were performed after a washout period of at least one month following completion of experimental procedures.

#### Statistical Analysis

Statistical analyses were performed using GraphPad Prism (version 8; GraphPad Software, San Diego, CA, USA) and Microsoft Excel (Microsoft Corporation, Redmond, WA, USA). Statistical significance was defined as *p* < 0.05. Data distribution was assessed for normality using the Shapiro–Wilk test. Homogeneity of variances was evaluated using the Brown–Forsythe test. Based on these assumptions, appropriate statistical tests were selected. Hence, when data met both normality and homoscedasticity assumptions, comparisons among groups were performed using ordinary one-way analysis of variance (one-way ANOVA). When data met the assumption of normality but violated homogeneity of variances, Welch’s ANOVA was applied. When data violated both normality and homoscedasticity assumptions, comparisons were performed using the non-parametric Kruskal–Wallis test. Post hoc multiple comparisons were conducted using Tukey’s test following parametric ANOVA, or Dunn’s test following Kruskal–Wallis test.

Data is expressed as mean ± standard error of the mean (SEM), or mean ± standard deviation (SD), if it meets normal distribution, if not, by interquartile range (IQR). As each experimental unit corresponds to a single animal, each data point represents an independent biological replicate.

## Results

### Cylinder Test

**Fig. 2:**
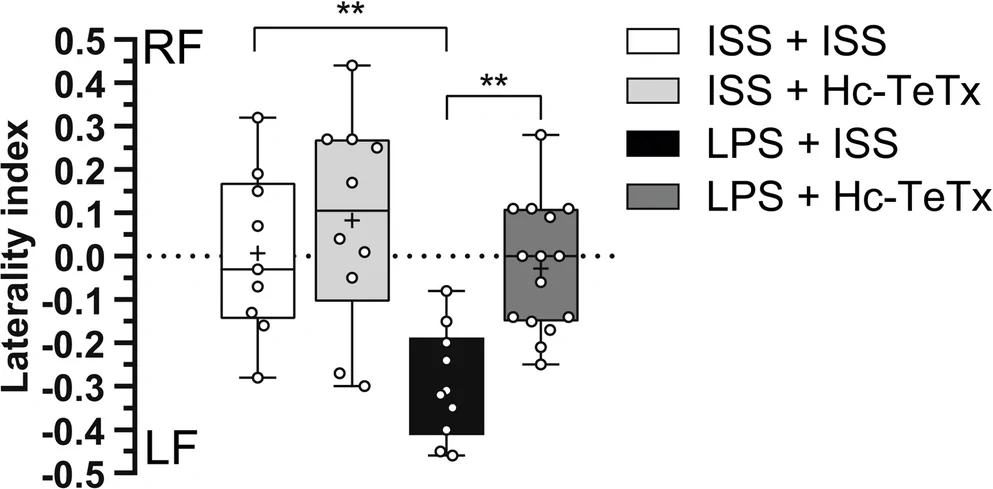
Effects of Hc-TeTx on Forelimb use asymmetry in LPS-induced parkinsonian model. Motor performance was evaluated 8 days after surgery using the cylinder test. LPS caused significant motor asymmetry that was normalized by Hc-TeTx treatment. Graph show the Laterality Index (LI) where positive index refers to Right forelimb (RF) use preference and negative index refers to Left forelimb (LF) use preference during rearing. ISS + ISS (*n* = 9), ISS + Hc-TeTx (*n* = 10), LPS + ISS (*n* = 10), LPS + Hc-TeTx (*n* = 15). *p* < 0.01 (**). Statistical analysis was performed with Ordinary One-way ANOVA test, followed by Tukey post hoc test. Each dot corresponds to a single experimental subject. Data is represented by box and whisker plots showing median, mean (+), IQR and range. Hc-TeTx: Recombinant C-terminal tetanus toxin; ISS, isotonic saline solution; LF, left forelimb; LI, Laterality index; LPS, lipopolysaccharide; RF, right forelimb

Forelimb use asymmetry during spontaneous vertical exploratory was evaluated using the cylinder test 8 days after stereotaxic surgery. Descriptive analysis showed that the LI was markedly reduced in the LPS + ISS group (− 0.30 ± 0.13) compared to the control group (ISS + ISS: 0.001 ± 0.19) (Fig. 2) and that Hc-TeTx treatment partially restored this parameter (LPS + Hc-TeTx (− 0.03 ± 0.15)).

One-way ANOVA revealed a significant effect of treatment on the LI (F(3,40) = 8.51, *p* < 0.001, R² = 0.390). Multiple comparisons test (Fig. 2) revealing that LPS caused a significant decrease in LI respect to control (mean difference = − 0.303, *p* < 0.01). These results indicate a marked shift toward contralateral FL use impairment following LPS administration. Importantly, the LI in the LPS + Hc-TeTx group was significantly higher compared to the LPS + ISS group (mean difference = 0.268, *p* < 0.01), indicating a restoration of FL motor function. No significant differences were observed between the ISS + ISS and ISS + Hc-TeTx groups, nor between the ISS + ISS and LPS + Hc-TeTx groups, suggesting that Hc-TeTx administration alone did not alter motor function in non-lesioned animals and restored motor performance toward control levels in LPS-lesioned animals.

Overall, these findings indicate that intracerebral LPS administration induced significant FL motor asymmetry consistent with unilateral dopaminergic dysfunction, and that Hc-TeTx treatment significantly attenuated this motor deficit.

### Elevated Beam Test

Motor coordination, balance, and gait performance were evaluated using the elevated beam test 7 days after LPS injection. Overall, no statistically significant differences were observed among experimental groups in any of the analyzed parameters (Table 1).

**Table 1:** Summary of gait parameters results

Variable	ISS + ISS	ISS + Hc-TeTx	LPS + ISS	LPS + Hc-TeTx
Latency (s)	4.42 (2.24)^a^	3.47 (1.35) ^a^	4.54 (2.71) ^a^	3.98 (1.82) ^a^
Velocity (cm/s)	25.34 (± 7.20)	29.43 (± 6.98)	25.78 (± 9.58)	25.24 (± 6.35)
Hindlimb (HL) strides	7.80 (1.60) ^a^	8.10 (1.40) ^a^	7.08 (3.80) ^a^	8.00 (1.60) ^a^
Forelimb (FL) strides	8.60 (2.00) ^a^	8.40 (1.60) ^a^	8.6 (6.20) ^a^	9.20 (2.40) ^a^
Cadence HL (stride/s)	1.94 (1.47) ^a^	2.98 (1.58) ^a^	2.08 (1.88) ^a^	2.12 (1.51) ^a^
Cadence FL (stride/s)	2.08 (1.61) ^a^	2.30 (1.74) ^a^	2.40 (1.72) ^a^	2.15 (1.64) ^a^
Step cycle HL (s)	0.46 (± 0.14)	0.40 (± 0.07)	0.39 (± 0.10)	0.40 (± 0.08)
Step cycle FL (s)	0.43 (± 0.11)	0.40 (± 0.09)	0.39 (± 0.10)	0.40 (± 0.07)
Stance time HL (%)	74.00 (± 6.23)	72.40 (± 4.59)	72.8 (± 5.42)	72.95 (± 5.62)
Swing time HL (%)	26.00 (± 6.28)	27.60 (± 4.60)	27.20 (± 5.42)	27.05 (± 5.62)
Stance time FL (%)	70.01 (± 9.14)	70.90 (± 4.57)	70.40 (± 5.79)	71.20 (± 5.57)
Swing time FL (%)	29.90 (± 9.14	29.10 (± 4.57)	29.60 (± 5.79)	28.80 (± 5.57)
Stride length HL (cm)	14.62 (± 2.88)	15.54 (± 2.74)	12.75 (± 3.69)	13.32 (± 2.85)
Stride length FL (cm)	14.30 (± 3.51)	15.53 (± 3.33)	13.10 (± 3.71)	12.08 (± 3.05)

Motor coordination, balance, and gait performance were evaluated using the elevated beam test 7 days after stereotaxic surgery. Overall, no statistically significant differences were observed among experimental groups in any of the analyzed parameters. ^a^ Values expressed as median (IQR) due to non-parametric analysis (Kruskal–Wallis test). All other values are expressed as mean (± SD) due to parametric analysis (one-way ANOVA). *Hc-TeTx* Recombinant C-terminal domain of the heavy-chain of tetanus toxin, *FL* forelimb, *HL* Hindlimb, *ISS* isotonic saline solution, *LF* left forelimb, *LPS* lipopolysaccharide, *RF* right forelimb

Analysis of beam traversal latency revealed comparable performance across groups, with median values ranging from 3.47 to 4.54 s. Similarly, mean velocity did not differ significantly among groups with mean from 25.1 to 29 cm/s, indicating preserved or compensatory locomotor capacity despite inflammatory insult. Spatial and temporal gait parameters, including the number of HL and FL strides, cadence, step cycle duration, stance time, swing time, and stride length, were also comparable among all experimental groups. These findings suggest that neither LPS administration nor Hc-TeTx treatment significantly altered basic locomotor rhythm or interlimb coordination under the present experimental conditions (Table 1).

**Fig. 3:**
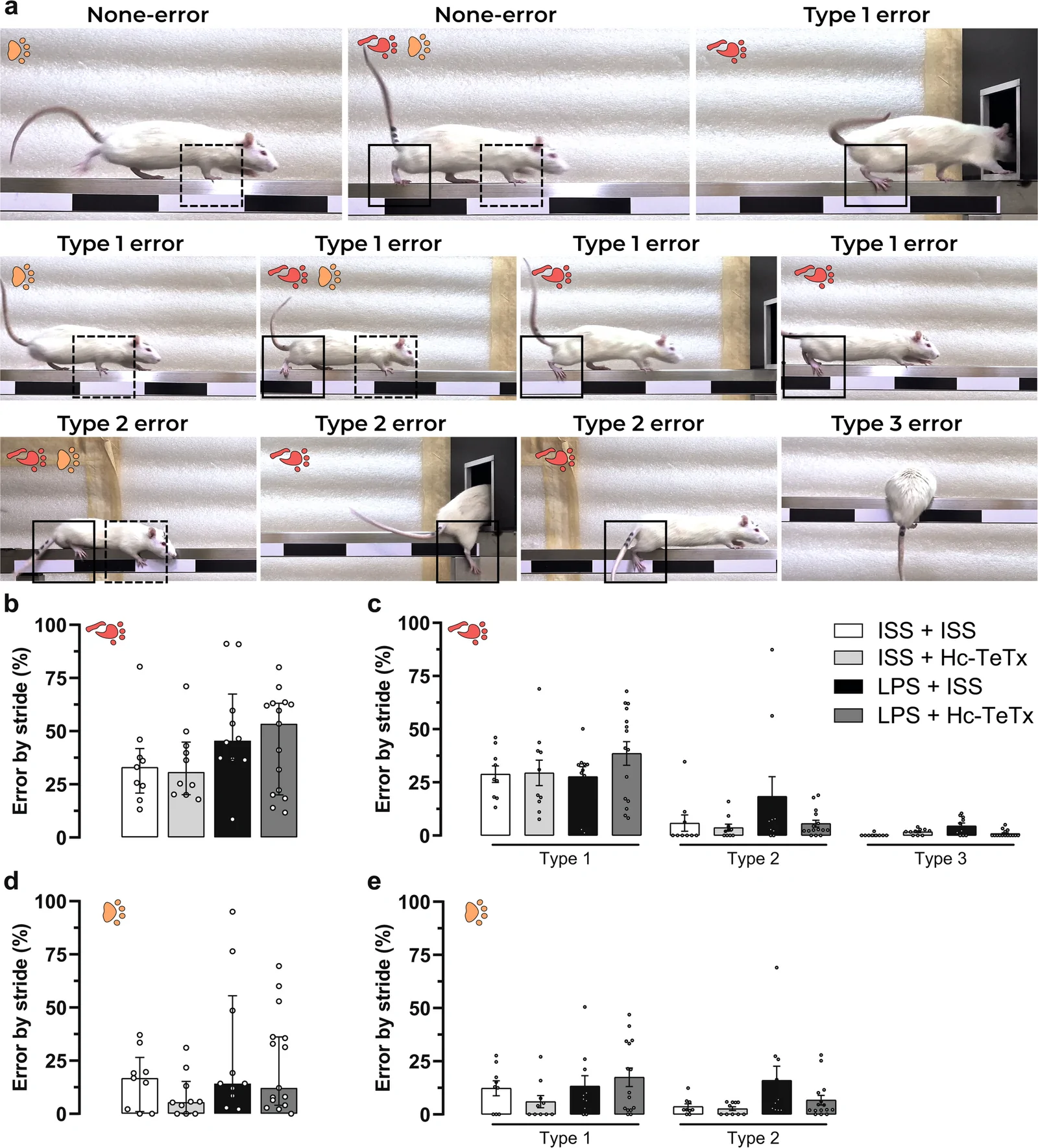
Effects of Hc-TeTx on motor function in LPS-induced parkinsonian model. Quadrupedal motor skills were evaluated 7 days after LPS injection using the elevated beam test. Neither LPS nor Hc-TeTx treatment significantly modified the Quadrupedal motor skills. Panel a depicts photographs that represent type of error in gait above beam. Panels b and d show errors by step proportion of contralateral FL and HL respectively. Panels c and e show the type of errors by step proportion of contralateral fore and hind limbs, respectively. ISS + ISS (*n* = 9), ISS + Hc-TeTx (*n* = 10), LPS + ISS (*n* = 10), LPS + Hc-TeTx (*n* = 15). Each dot corresponds to mean of five completed crosses by each experimental subject. Data in b and d are represented as median and IQR, and in c and e are presented as mean ± SEM. Statistical analysis was performed with Kruskal–Wallis test followed by post Dunn’s test for b and d and Two-way ANOVA test for c and e, followed by post Tukey’s multiple comparison test. Hc-TeTx, Recombinant C-terminal domain of the heavy-chain of tetanus toxin; ISS, isotonic saline solution; LPS, lipopolysaccharide

Two-way ANOVA revealed a significant main effect of error type (F(2,120) = 53.31, *p* < 0.001), indicating that the frequency of stepping errors differed across error categories. However, no significant main effect of treatment (F(3,120) = 1.082, *p* = 0.359) or interaction between treatment and error type (F(6,120) = 1.666, *p* = 0.135) was observed. Motor accuracy, assessed by the percentage of step errors also did not differ significantly among groups. Although descriptive analysis showed a trend toward higher HL error rates in LPS-treated animals compared to control groups (LPS + ISS: median 45.5%, IQR 30.6; LPS + Hc-TeTx: median 53.5%, IQR 43.1), these differences did not reach statistical significance (Fig. 3). Similarly, FL error rates and the relative proportion of error types remained statistically comparable among all experimental groups.

Together, these findings indicate that unilateral LPS administration did not produce measurable impairments in beam walking performance at the evaluated time point. Furthermore, Hc-TeTx treatment did not significantly modify motor performance in either control or LPS-lesioned animals. These results suggest that the elevated beam test may be less sensitive than the cylinder test for detecting early or moderate unilateral motor deficits induced by neuroinflammatory insult.

### Dopaminergic Neuron Survival in the Substantia Nigra Pars Compacta

**Fig. 4:**
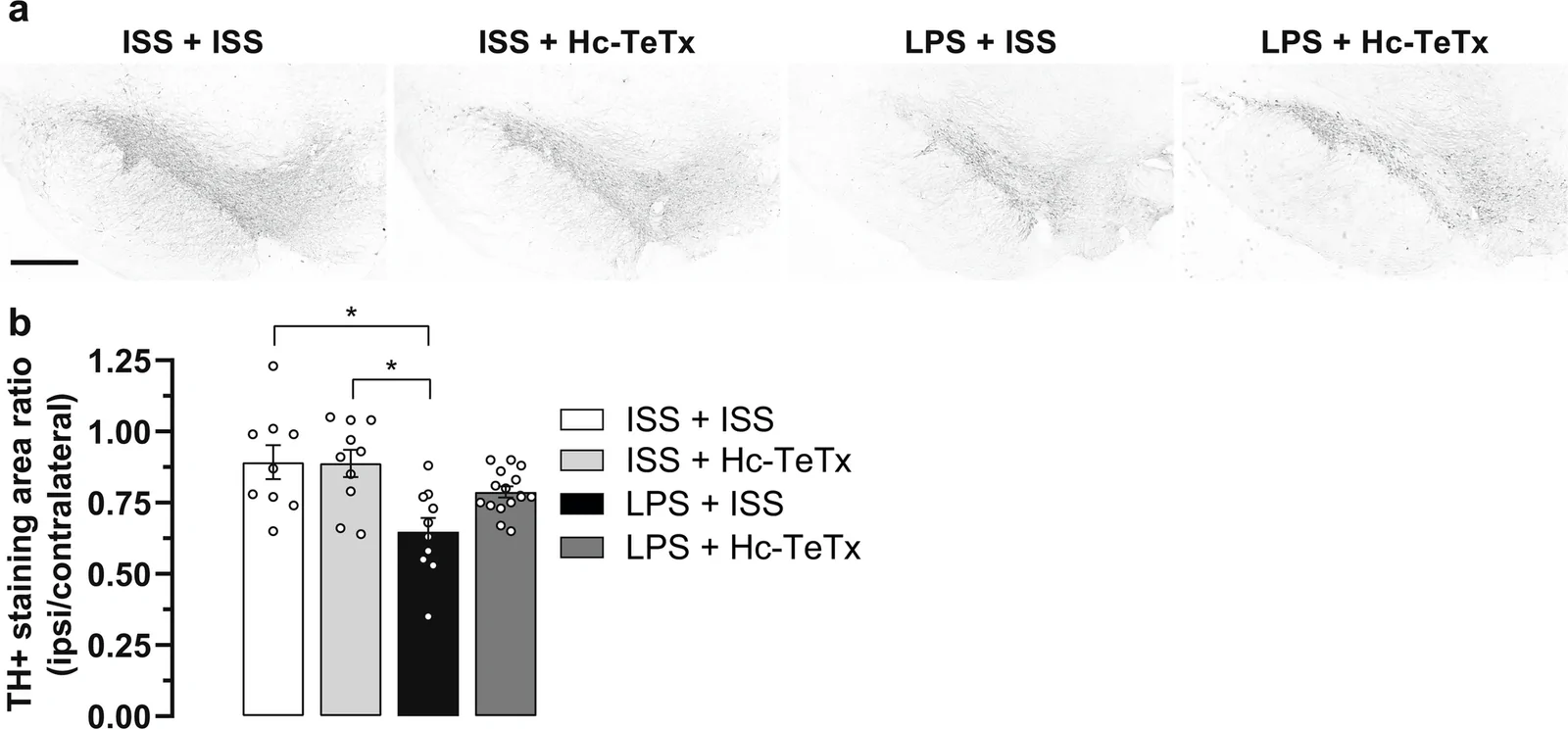
Effects of Hc-TeTx on neurodegeneration in LPS-induced parkinsonian model. Neurodegeneration was evaluated by immunohistochemical (IHC) staining after euthanasia. Hc-TeTx treatment conferred partial neuroprotection to dopaminergic neurons in *SNpc*. (a) micrographs panel of representative TH+ neurons on SNpc (10x; approximately − 5.0 mm from bregma). (b) ratio of ipsilateral/contralateral hemispheres of TH+ neurons in the *SNpc*. ISS + ISS (*n* = 9), ISS + Hc-TeTx (*n* = 10), LPS + ISS (*n* = 10), LPS + Hc-TeTx (*n* = 15). *p* < 0.05 (*). Scale bar: 250 μm. Each dot corresponds to each experimental subject. Data is presented as mean ± SEM. Statistical analysis was performed with Ordinary One-way ANOVA test, followed by post Tukey’s multiple comparison test. Hc-TeTx: Recombinant C-terminal domain of the heavy-chain of tetanus toxin; ISS, isotonic saline solution; LPS, lipopolysaccharide; TH+, tyrosine hydroxylase positive cell

Dopaminergic neurodegeneration was assessed by quantifying the ratio of TH+ neurons between the ipsilateral and contralateral hemispheres in the *SNpc.* Descriptive analysis confirmed a marked reduction in TH+ neuron survival in the LPS + ISS group (0.65 ± 0.16) compared to control groups (ISS + ISS: 0.89 ± 0.17; ISS + Hc-TeTx: 0.89 ± 0.15), whereas Hc-TeTx treatment increased this ratio to 0.78 ± 0.08 in LPS group (Fig. 4).

One-way ANOVA revealed a significant effect of treatment on TH+ neuron (F(3,40) = 6.82, *p* < 0.001, R² = 0.338), indicating that LPS administration and Hc-TeTx treatment differentially affected dopaminergic neuron integrity. Post hoc comparisons using Tukey’s test demonstrated that animals in the LPS + ISS group exhibited a significantly lower TH ipsilateral/contralateral ratio compared to both ISS + ISS group (mean difference = − 0.244, *p* < 0.01) and ISS + Hc-TeTx group (mean difference = − 0.240, *p* < 0.01). These findings confirm that intracerebral LPS administration induced significant dopaminergic neurodegeneration in the *SNpc*. Importantly, treatment with Hc-TeTx alone did not affect dopaminergic neurons, however, it did at least partially attenuate LPS-induced dopaminergic neuron loss. Although the TH ratio was higher in the LPS + Hc-TeTx group (0.78 ± 0.08) compared to the LPS + ISS group (0.65 ± 0.16), this difference did not reach statistical significance (mean difference = 0.139, *p* = 0.079). Nevertheless, the TH ratio in the LPS + Hc-TeTx group was not significantly different from either control group (ISS + ISS: *p* = 0.285; ISS + Hc-TeTx: *p* = 0.292), suggesting a partial restoration of dopaminergic neuron integrity toward baseline levels. No significant differences were observed between the ISS + ISS and ISS + Hc-TeTx groups, indicating that Hc-TeTx administration alone did not affect dopaminergic neuron in the absence of inflammatory insult.

Overall, these findings demonstrate that intrastriatal LPS administration induces significant dopaminergic neurodegeneration in the SNpc, and that Hc-TeTx treatment confers partial neuroprotection against inflammation-induced neuronal loss.

### Astroglial Activation in the Striatum

**Fig. 5:**
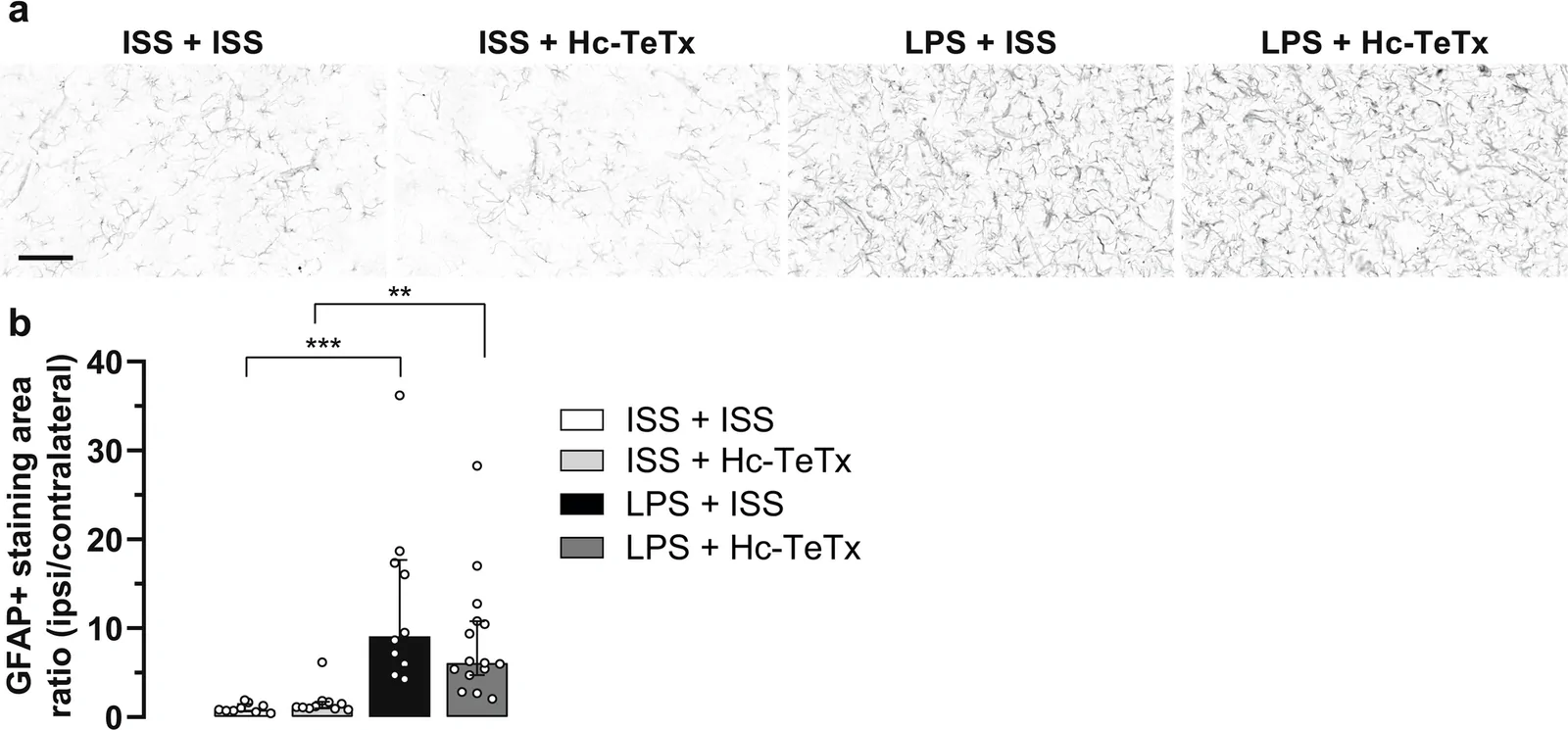
Effects of Hc-TeTx on Astrogliosis in LPS-induced parkinsonian model. Astrogliosis was evaluated by immunohistochemical (IHC) staining. The reduction in LPS-induced astrogliosis in the striatum by Hc-TeTx did not reach statistical significance. (a) micrographs panel of representative GFAP+ cells on striatum (20x; approximately 0.8 mm from bregma). (b) ratio of ipsilateral/contralateral hemispheres for the number of GFAP+ cells in the striatum. ISS + ISS (*n* = 9), ISS + Hc-TeTx (*n* = 10), LPS + ISS (*n* = 10), LPS + Hc-TeTx (*n* = 15). *p* < 0.01 (**), *p* < 0.001 (***). Scale bar: 100 μm. Each dot corresponds to each experimental subject. Data is presented as median and IQR. Statistical analysis was performed with Kruskal-Wallis test, followed by post Dunn’s multiple comparison test. Hc-TeTx: Recombinant C-terminal domain of the heavy-chain of tetanus toxin; ISS, isotonic saline solution; LPS, lipopolysaccharide; GFAP+, glial fibrillar acid protein-positive

Astrogliosis was evaluated by quantifying the ratio of GFAP-immunoreactive area between the ipsilateral and contralateral hemispheres in the dorsolateral striatum. Descriptive analysis showed that the GFAP ipsilateral/contralateral ratio was markedly increased in LPS-treated animals (LPS + ISS: median 9.09, IQR 11.34; LPS + Hc-TeTx: median 6.11, IQR 6.06) compared to control groups (ISS + ISS: median 0.85, IQR 0.8; ISS + Hc-TeTx: median 1.18, IQR 0.737) (Fig. 5).

Statistical analysis using the Kruskal–Wallis test revealed a significant effect of treatment on GFAP immunoreactivity (H = 30.1, *p* < 0.001). Post hoc analysis using Dunn’s multiple comparisons test demonstrated a significant increase in GFAP immunoreactivity in the LPS-treatment animals compared to both control groups (ISS + ISS: *p* < 0.001; ISS + Hc-TeTx: *p* < 0.01 [not showed to simplify graph]). Similarly, animals in the LPS + Hc-TeTx group also exhibited significantly higher GFAP ratios compared to the and the control groups ISS + Hc-TeTx group (*p* < 0.01) and ISS + ISS group (*p* < 0.001, [not showed to simplify graph]), confirming robust astroglial activation following LPS administration. No significant differences were observed between the ISS + ISS and ISS + Hc-TeTx groups, indicating that Hc-TeTx treatment alone did not induce astroglial activation. Importantly, median GFAP ratio was lower in the LPS + Hc-TeTx (6.11, 6.06) group compared to the LPS + ISS group (9.09, 0.8), though this difference did not reach statistical significance (*p* > 0.999).

These findings indicate that intracerebral LPS administration induced pronounced astroglial activation in the striatum, and that Hc-TeTx treatment showed a tendency to reduce this astrocytosis but did not significantly prevent or reverse inflammation-associated astrogliosis under the present experimental conditions.

## Discussion

Out findings demonstrate that prophylactic administration of recombinant Hc-TeTx significantly attenuates LPS-induced FL motor asymmetry during spontaneous vertical exploration in the cylinder test and partially preserves dopaminergic neurons in a neuroinflammatory model of PD. These findings provide novel evidence supporting the functional neuroprotective effects of Hc-TeTx in a model in which neurodegeneration is initiated and sustained by inflammation rather than by direct neurotoxic insult. This distinction is particularly relevant, as accumulating evidence indicates that neuroinflammation is not a secondary phenomenon in PD, but a central driver of disease initiation and progression (Hirsch and Hunot 2009; Gagne and Power 2010; Castillo-Rangel et al. 2023; Chen et al. 2026).

Hc-TeTx has been shown to undergo retrograde transport and activate intracellular survival pathways, including PI3K/Akt and MAPK signaling cascades, which promote neuronal survival and functional integrity (Gil et al. 2000, 2003; Chaïb-Oukadour et al. 2004). However, evidence directly linking Hc-TeTx to modulation of inflammatory pathways remains limited. Available studies indicate that Hc-TeTx can reduce TNF-α expression in the depression-prone Wistar-Kyoto rats (Getachew et al. 2019), decrease NLRP3 inflammasome components in amyotrophic lateral sclerosis model (Moreno-Martinez et al. 2020), and attenuate microgliosis and astrogliosis in toxin-induced neurodegeneration paradigms (Mendieta et al. 2012, 2016; Patricio et al. 2019, 2022). These observations suggest that Hc-TeTx may influence inflammatory responses under certain pathological conditions, although the exact neuromodulatory effects of this compound remain to be determined.

Interestingly, in the present model, Hc-TeTx attenuation of the astroglial activation did not achieve statistical significance. This finding underscores the difference between toxin-based models, in which glial activation is believed to arise secondary to neuronal damage. In contrast, it is believed that LPS directly activates innate immune signaling pathways, leading to sustained glial reactivity that may persist independently of neuronal survival. Moreover, reactive astrocytes and microglia are not exclusively deleterious as under specific contexts, they may contribute to containment of tissue damage and structural remodeling rather than actively promoting degeneration (Teismann and Schulz 2004; Hirsch and Hunot 2009; Parra et al. 2020; Tian et al. 2026).

The finding that Hc-TeTx produced robust functional protection but only partial preservation of dopaminergic neurons, suggests that its neuroprotective effects may occur independently of overt suppression of glial activation. Moreover, contributions from inflammatory propagation affecting neighboring or functionally interconnected basal ganglia regions cannot be ruled out. Thus, rather than acting as a broad anti-inflammatory agent, Hc-TeTx may enhance intrinsic neuronal resilience to inflammatory stress through activation of survival signaling pathways, thereby preserving functional output even in the presence of sustained glial reactivity. Preservation of motor function in this inflammatory context highlights the capacity of Hc-TeTx to maintain circuit-level integrity under sustained neuroimmune challenge.

An important limitation of the present study is that Hc-TeTx was administered as a single prophylactic dose prior to LPS exposure. Although this approach allowed assessment of its protective potential under controlled experimental conditions, it does not fully reproduce the clinical scenario in which therapeutic intervention occurs after disease onset. Therefore, longitudinal studies using delayed therapeutic administration and repeated treatment paradigms after injury induction will be essential to better evaluate translational relevance. Future studies should also investigate the molecular mechanisms underlying Hc-TeTx-mediated neuroprotection, including whether modulation of microglial activation, astroglial responses, inflammatory cytokines, oxidative stress pathways, or inflammasome signaling contributes to the observed effects. Such studies will help determine whether Hc-TeTx exerts direct or indirect immunomodulatory actions. This is noteworthy as the present study was not designed to establish whether the observed neuroprotective effects are mediated by immunomodulatory mechanisms, rather to provide proof of the concept that Hc-TeTx can affect an inflammatory model of PD. Thus, the above-mentioned studies are necessary to provide a more comprehensive mechanistic understanding of the therapeutic potential of Hc-TeTx in inflammation-associated neurodegeneration.

In summary, Hc-TeTx significantly improves motor asymmetry and partially preserves dopaminergic neuron survival in an ongoing inflammation-driven model of PD. These findings extend previous evidence of its neuroprotective properties to a context in which neuroinflammation is the primary pathogenic driver and provides further support for considering Hc-TeTx as a therapeutic strategy in an inflammatory PD model.

## Data Availability

The datasets used and/or analyzed during the current study will be available from the corresponding author on reasonable request.

## Abbreviations

ANOVA: Analysis of variance
AP: Anteroposterior
BF: Both forelimbs
*CCUAL*: *Comité para el Cuidado y Uso de los Animales de Laboratorio*
DAB: 3,3′-diaminobenzidine tetrahydrochloride hydrate
DV: Dorsoventral
FL: Forelimb
GFAP: Glial fibrillary acidic protein
GFAP+: GFAP-positive cells or immunoreactivity
Hc‑TeTx: C-terminal domain of the heavy chain of tetanus toxin
HL: Hindlimbs
HRP: horseradish peroxidase
i.c.: Intracranial
i.p.: Intraperitoneal
IHC: Immunohistochemistry
IQR: Interquartile range
LF: Left forelimb
LI: Laterality index
LPS: Lipopolysaccharide
MAPK: Mitogen-activated protein kinase
ML: Mediolateral
MPP+: 1-methyl-4-phenylpyridinium
MPTP: 1-methyl-4-phenyl-1,2,3,6-tetrahydropyridine
NLRP3: NOD-, LRR- and pyrin domain-containing protein 3
NOM: *Norma Oficial Mexicana*
PBS: Phosphate-buffered saline
PD: Parkinson’s disease
PI3K/Akt: Phosphoinositide 3-kinase/Protein kinase B signaling pathway
RF: Right forelimb
SD: Standard deviation
SEM: Standard error of the mean
*SN*: *Substantia nigra*
*SNpc*: *Substantia nigra pars compacta*
ISS: Isotonic saline solution
TH: Tyrosine hydroxylase
TH+: TH-positive neurons or immunoreactivity
6-OHDA: 6-hydroxydopamine
