# Correction to: C-terminal Domain of the Heavy Chain of Tetanus Toxin Ameliorate Lipopolysaccharide Induced Hemiparkinsonism in Rats

**DOI:** 10.1007/s12640-026-00814-1

**Published:** 2026-07-29

**Authors:** Irving Parra, José Aguilera, Yousef Tizabi, Liliana Mendieta

**Affiliations:** 1 Laboratorio de Neuroquímica, Facultad de Ciencias Químicas, Benemérita Universidad Autónoma de Puebla, 14 Sur y Av. San Claudio CU, Col. San Manuel, Puebla, 72592 México; 2 Departament de Bioquímica i Biologia Molecular, Institut de Neurociències, Universitat Autònoma de Barcelona, Cerdanyola del Vallès, Barcelona, 08193 España; 3 Centro de Investigación Biomédica en Red Enfermedades Neurodegenerativas (CIBERNED), Instituto de Salud Carlos III, Madrid, 28031 España; 4 Department of Pharmacology, Howard University College of Medicine, Washington, DC USA

**Correction to: Neurotoxicity Research (2026) 44:36**

**https://doi.org/10.1007/s12640-026-00811-4**

The original published version of this article unfortunately contained an error.

In the published article, the figure captions for Figures 1-5 were inadvertently placed in the main text by the production team.

The correct Figures 1-5 captions should have been presented as follows:

**Fig. 1** Timeline of the experimental design. Intracerebral injections of lipopolysaccharide (LPS) were performed in the left dorsolateral striatum of male rats (Rattus norvegicus; 291 ± 18 g at the time of surgery; *N* = 44) according to Paxinos and Watson (2014) coordinates. Experimental subjects performing behavioral test as Cylinder Test and Beam Walking Test to evaluated forelimb motor asymmetry during spontaneous vertical exploration and gait parameters and motor coordination, respectively. Animals were euthanized 8 days after surgery. Brains was processed for immunohistochemistry. Hemi-parkinsonian model induction: LPS [16 μg/μL] or LPS’ vehicle (isotonic saline solution, ISS) was injected stereotactically on two dorsoventral coordinates each one on striatum at AP: +0.8 mm; ML: +3.8 mm; DV1: −4.9 mm; DV2: −5.3 mm. Treatment regimen: Recombinant C terminal domain of heavy chain tetanus toxin (Hc-TeTx) or vehicle treatment ISS was administered one day before to stereotaxic surgery administered intraperitoneally. Abbreviations: Hc-TeTx, Recombinant C terminal domain of heavy chain of tetanus toxin; i.c, intracerebral administration; ISS, isotonic saline solution; LPS, lipopolysaccharide

**Fig. 2** Effects of Hc-TeTx on Forelimb use asymmetry in LPS-induced parkinsonian model. Motor performance was evaluated 8 days after surgery using the cylinder test. LPS caused significant motor asymmetry that was normalized by Hc-TeTx treatment. Graph show the Laterality Index (LI) where positive index refers to Right forelimb (RF) use preference and negative index refers to Left forelimb (LF) use preference during rearing. ISS + ISS (*n* = 9), ISS + Hc-TeTx (*n* = 10), LPS + ISS (*n* = 10), LPS + Hc-TeTx (*n* = 15). *p* < 0.01 (**). Statistical analysis was performed with Ordinary One-way ANOVA test, followed by Tukey post hoc test. Each dot corresponds to a single experimental subject. Data is represented by box and whisker plots showing median, mean (+), IQR and range. Hc-TeTx: Recombinant C-terminal tetanus toxin; ISS, isotonic saline solution; LF, left forelimb; LI, Laterality index; LPS, lipopolysaccharide; RF, right forelimb

**Fig. 3** Effects of Hc-TeTx on motor function in LPS-induced parkinsonian model. Quadrupedal motor skills were evaluated 7 days after LPS injection using the elevated beam test. Neither LPS nor Hc-TeTx treatment significantly modified the Quadrupedal motor skills. Panel a depicts photographs that represent type of error in gait above beam. Panels b and d show errors by step proportion of contralateral FL and HL respectively. Panels c and e show the type of errors by step proportion of contralateral fore and hind limbs, respectively. ISS + ISS (*n* = 9), ISS + Hc-TeTx (*n* = 10), LPS + ISS (*n* = 10), LPS + Hc-TeTx (*n* = 15). Each dot corresponds to mean of five completed crosses by each experimental subject. Data in b and d are represented as median and IQR, and in c and e are presented as mean ± SEM. Statistical analysis was performed with Kruskal–Wallis test followed by post Dunn’s test for b and d and Two-way ANOVA test for c and e, followed by post Tukey’s multiple comparison test. Hc-TeTx, Recombinant C-terminal domain of the heavy-chain of tetanus toxin; ISS, isotonic saline solution; LPS, lipopolysaccharide

**Fig. 4** Effects of Hc-TeTx on neurodegeneration in LPS-induced parkinsonian model. Neurodegeneration was evaluated by immunohistochemical (IHC) staining after euthanasia. Hc-TeTx treatment conferred partial neuroprotection to dopaminergic neurons in *SNpc*. (a) micrographs panel of representative TH+ neurons on SNpc (10x; approximately − 5.0 mm from bregma). (b) ratio of ipsilateral/contralateral hemispheres of TH+ neurons in the *SNpc*. ISS + ISS (*n* = 9), ISS + Hc-TeTx (*n* = 10), LPS + ISS (*n* = 10), LPS + Hc-TeTx (*n* = 15). *p* < 0.05 (*). Scale bar: 250 μm. Each dot corresponds to each experimental subject. Data is presented as mean ± SEM. Statistical analysis was performed with Ordinary One-way ANOVA test, followed by post Tukey’s multiple comparison test. Hc-TeTx: Recombinant C-terminal domain of the heavy-chain of tetanus toxin; ISS, isotonic saline solution; LPS, lipopolysaccharide; TH+, tyrosine hydroxylase positive cell

**Fig. 5** Effects of Hc-TeTx on Astrogliosis in LPS-induced parkinsonian model. Astrogliosis was evaluated by immunohistochemical (IHC) staining. The reduction in LPS-induced astrogliosis in the striatum by Hc-TeTx did not reach statistical significance. (a) micrographs panel of representative GFAP+ cells on striatum (20x; approximately 0.8 mm from bregma). (b) ratio of ipsilateral/contralateral hemispheres for the number of GFAP+ cells in the striatum. ISS + ISS (*n* = 9), ISS + Hc-TeTx (*n* = 10), LPS + ISS (*n* = 10), LPS + Hc-TeTx (*n* = 15). *p* < 0.01 (**), *p* < 0.001 (***). Scale bar: 100 μm. Each dot corresponds to each experimental subject. Data is presented as median and IQR. Statistical analysis was performed with Kruskal-Wallis test, followed by post Dunn’s multiple comparison test. Hc-TeTx: Recombinant C-terminal domain of the heavy-chain of tetanus toxin; ISS, isotonic saline solution; LPS, lipopolysaccharide; GFAP+, glial fibrillar acid protein-positive

This error does not affect the results, discussion, or conclusions of the article.

The original article has been corrected.

